# A two-component protein condensate of the EGFR cytoplasmic tail and Grb2 regulates Ras activation by SOS at the membrane

**DOI:** 10.1073/pnas.2122531119

**Published:** 2022-05-04

**Authors:** Chun-Wei Lin, Laura M. Nocka, Brittany L. Stinger, Joseph B. DeGrandchamp, L. J. Nugent Lew, Steven Alvarez, Henry T. Phan, Yasushi Kondo, John Kuriyan, Jay T. Groves

**Affiliations:** ^a^Department of Chemistry, University of California, Berkeley, CA 94720;; ^b^Department of Molecular and Cell Biology, University of California, Berkeley, CA 94720;; ^c^California Institute for Quantitative Biosciences, University of California, Berkeley, CA 94720;; ^d^HHMI, Chevy Chase, MD 20815;; ^e^Division of Molecular Biophysics and Integrated Bioimaging, Lawrence Berkeley National Laboratory, Berkeley, CA 94720;; ^f^Institute for Digital Molecular Analytics and Science, Nanyang Technological University, 639798 Singapore

**Keywords:** protein condensate, condensation phase transition, EGFR, membrane, Ras

## Abstract

We reconstitute a phosphotyrosine-mediated protein condensation phase transition of the ∼200 residue cytoplasmic tail of the epidermal growth factor receptor (EGFR) and the adaptor protein, Grb2, on a membrane surface. The phase transition depends on phosphorylation of the EGFR tail, which recruits Grb2, and crosslinking through a Grb2-Grb2 binding interface. The Grb2 Y160 residue plays a structurally critical role in the Grb2-Grb2 interaction, and phosphorylation or mutation of Y160 prevents EGFR:Grb2 condensation. By extending the reconstitution experiment to include the guanine nucleotide exchange factor, SOS, and its substrate Ras, we further find that the condensation state of the EGFR tail controls the ability of SOS, recruited via Grb2, to activate Ras. These results identify an EGFR:Grb2 protein condensation phase transition as a regulator of signal propagation from EGFR to the MAPK pathway.

## Significance

Two-dimensional condensates of proteins on the membrane surface, driven by tyrosine phosphorylation, are beginning to emerge as important players in signal transduction. This work describes discovery of a protein condensation phase transition of EGFR and Grb2 on membrane surfaces, which is poised to have a significant impact on how we understand EGFR signaling and misregulation in disease. EGFR condensation is mediated through a Grb2-Grb2 crosslinking element, which itself is regulatable through a specific phosphotyrosine site on Grb2. Furthermore, the EGFR condensate exerts significant control over the ability of SOS to activate Ras, thus implicating the EGFR condensate as a regulator of signal propagation from EGFR to Ras and the MAPK pathway.

Recently, a class of phenomena known as protein condensation phase transitions has begun to emerge in biology. Originally identified in the context of nuclear organization (1) and gene expression (2), a distinct two-dimensional protein condensation on the cell membrane has now been discovered in the T cell receptor (TCR) signaling system involving the scaffold protein LAT (3–5). TCR activation results in phosphorylation of LAT on at least four distinct tyrosine sites, which subsequently recruit the adaptor protein Grb2 and the signaling molecule PLCγ via selective binding interactions with their SH2 domains. Additional scaffold and signaling molecules, including SOS, GADS, and SLP76, are recruited to Grb2 and PLCγ through further specific protein–protein interactions (6, 7). Multivalency among some of these binding interactions can crosslink LAT molecules in a two-dimensional bond percolation network on the membrane surface. The resulting LAT protein condensate resembles the nephrin:NCK:*N*-WASP condensate (8) in that both form on the membrane surface under control of tyrosine phosphorylation and exert at least one aspect of functional control over signaling output via a distinct type of kinetic regulatory mechanism (9–11). The basic molecular features controlling the LAT and nephrin protein condensates are common among biological signaling machinery, and other similar condensates continue to be discovered (12, 13). The LAT condensation shares downstream signaling molecules with the EGF-receptor (EGFR) signaling system, raising the question if EGFR may participate in a signaling-mediated protein condensation itself.

EGFR signals to the mitogen-activated protein kinase (MAPK) pathway and controls key cellular functions, including growth and proliferation (14–16). EGFR is a paradigmatic model system in studies of signal transduction, and immense, collective scientific effort has revealed the inner workings of its signaling mechanism down to the atomic level (17). EGFR is autoinhibited in its monomeric form. Ligand-driven activation is achieved through formation of an asymmetric receptor dimer in which one kinase activates the other to phosphorylate the nine tyrosine sites in the C-terminal tails (17, 18). There is an obvious conceptual connection between EGFR and the LAT signaling system in T cells. The ∼200-residue–long cytoplasmic tail of EGFR resembles LAT in that both are intrinsically disordered and contain multiple sites of tyrosine phosphorylation that recruit adaptor proteins, including Grb2, upon receptor activation (19). Phosphorylation at tyrosine residues Y1068, Y1086, Y1148, and Y1173 in the EGFR tail creates sites to which Grb2 can bind via its SH2 domain. EGFR-associated Grb2 subsequently recruits SOS, through binding of its SH3 domains to the proline-rich domain of SOS. Once at the membrane, SOS undergoes a multistep autoinhibition-release process and begins to catalyze nucleotide exchange of RasGDP to RasGTP, activating Ras and the MAPK pathway (20).

While these most basic elements of the EGFR activation mechanism are widely accepted, larger-scale features of the signaling complex remain enigmatic. A number of studies have reported higher-ordered multimers of EGFR during activation, including early observations by Förster Resonance Energy Transfer and fluorescence lifetime studies (21–23), as have more recent studies using single molecule (24, 25) and computational methods (26). Structural analyses and point mutation studies on EGFR have identified a binding interface enabling EGFR asymmetric dimers to associate (27), but the role of these higher-order assemblies remains unclear. At the same time, many functional properties of the signaling system remain unexplained as well. For example, EGFR is a frequently altered oncogene in human cancers, and drugs (including tyrosine kinase inhibitors) targeting EGFR signaling have produced impressive initial patient responses (28). All too often, however, these drugs fail to offer sustained patient benefits, in large part because of poorly understood resistance mechanisms (29). Physical aspects of the cellular microenvironment have been implicated as possible contributors to resistance development (30), and there is a growing realization that EGFR possesses kinase-independent (e.g., signaling independent) prosurvival functions in cancer cells (31). These points fuel speculation that additional layers of regulation over the EGFR signaling mechanism exist, including at the level of the receptor signaling complex itself.

Here we report that EGFR undergoes a protein condensation-phase transition upon activation. We reconstituted the cytoplasmic tails of EGFR on supported bilayers and characterized the system behavior upon interaction with Grb2 and SOS, using total internal reflection fluorescence (TIRF) imaging. This experimental platform has been highly effective for revealing both phase-transition characteristics and functional signaling aspects of LAT protein condensates (4, 5, 10, 32–34). Published reports on the LAT system to date have emphasized SOS (or the SOS proline-rich [PR] domain) as a critical crosslinking element. Titrating the SOS PR domain into an initially homogeneous mixture of phosphorylated LAT and Grb2 revealed a sharp transition to the condensed phase, which we have also observed with the EGFR:Grb2:SOS system. Under slightly different conditions, however, we report observations of an EGFR:Grb2 condensation-phase transition without any SOS or other crosslinking molecule. We show that crosslinking is achieved through a Grb2–Grb2 binding interface. Phosphorylation on Grb2 at Y160 as well as a Y160E mutation [both reported to disrupt Grb2–Grb2 binding (35, 36)] were observed to prevent formation of EGFR condensates. We note that the evidence of Grb2–Grb2 binding we observed occurred in the context of EGFR-associated Grb2, which is localized to the membrane surface; free Grb2 dimers are not necessary.

The consequence of EGFR condensation on downstream signaling is characterized by mapping the catalytic efficiency of SOS to activate Ras as a function of the EGFR condensation state. SOS is the primary Ras guanine nucleotide exchange factor (GEF) responsible for activating Ras in the EGFR-to-MAPK signaling pathway (37–40). At the membrane, SOS undergoes a multistep process of autoinhibition release before beginning to activate Ras. Once fully activated, SOS is highly processive, and a single SOS molecule can activate hundreds of Ras molecules before disengaging from the membrane (41–43). Autoinhibition release in SOS is a slow process, which necessitates that SOS be retained at the membrane for an extended time in order for Ras activation to begin (5, 10). This delay between initial recruitment of SOS and subsequent initiation of its Ras GEF activity provides a kinetic proofreading mechanism that essentially requires SOS to achieve multivalent engagement with the membrane (e.g., through multiple Grb2 or other interactions) in order for it to activate any Ras molecules.

Experimental results described here reveal that Ras activation by SOS is strongly enhanced by EGFR condensation. Calibrated measurements of both SOS recruitment and Ras activation confirmed enhanced SOS catalytic activity on a per-molecule basis, in addition to enhanced recruitment to the condensates. These results suggest that a Grb2-mediated EGFR protein condensation-phase transition is a functional element controlling signal propagation from EGFR downstream to the MAPK signaling pathway.

## Results

### Reconstitution of a Membrane-Surface EGFR:Grb2 Protein Condensate.

We reconstituted an EGFR:Grb2 protein condensation-phase transition on supported membranes using the C-terminal region of EGFR (residues 991 to 1186, where the signal peptide of EGFR is not included in numbering), which is expressed along with a His6 tag and a SUMO tag at the N-terminal end of the construct. Throughout this article, we refer to this construct of the cytoplasmic tail of EGFR as the EGFR^TAIL^. EGFR^TAIL^ was anchored on the supported lipid bilayer through binding between its His tag and nickel–nitrilotriacetic acid (Ni-NTA) lipid (incorporated into the supported bilayer at a 4% molar ratio with 1,2-dioleoyl-sn-glycero-3-phosphocholine (DOPC) lipid as the primary component), following previously published methods (5, 10). The small ubiquitin-related modifier (SUMO) fusion tag was used to increase the expression yield of the disordered EGFR^TAIL^ and acted as the spacer between the surface of the bilayer and the tail of EGFR, where the native kinase domain of EGFR would normally be positioned. The EGFR^TAIL^ was fluorescently labeled with Alexa Fluor 488 through maleimide chemistry, and we achieved 66% labeling efficiency. EGFR^TAIL^ constructs anchored to the supported membrane underwent simple Brownian motion (D = 1.76 µm^2^/s, measured by the fluorescence recovery after photobleaching experiments; *SI Appendix*, Fig. S1) and appeared to be uniformly distributed on the membrane surface under TIRF microscopy. The lateral density of the EGFR^TAIL^ on the membrane can be precisely measured by fluorescence correlation spectroscopy (FCS) (5, 44) and was controlled to between 50 and 3000 molecules/µm^2^ in the experiments presented here. To maintain the EGFR^TAIL^ in its active, phosphorylated state, a separate Src-family kinase protein, Hck, was tethered to the membrane (also through His tag chemistry) and adenosine triphosphate (ATP) was provided in solution. Src kinases do not normally phosphorylate the EGFR^TAIL^ efficiently (19), but in this system, the preincubation with Hck sufficed to ensure robust phosphorylation of the tail. A similar strategy was used previously to maintain LAT in its phosphorylated state for condensation phase transition studies (5, 33, 45). Grb2 and, in later experiments, SOS were added to the prephosphorylated EGFR on supported membranes to initiate the condensation phase transition.

**Fig. 1.**
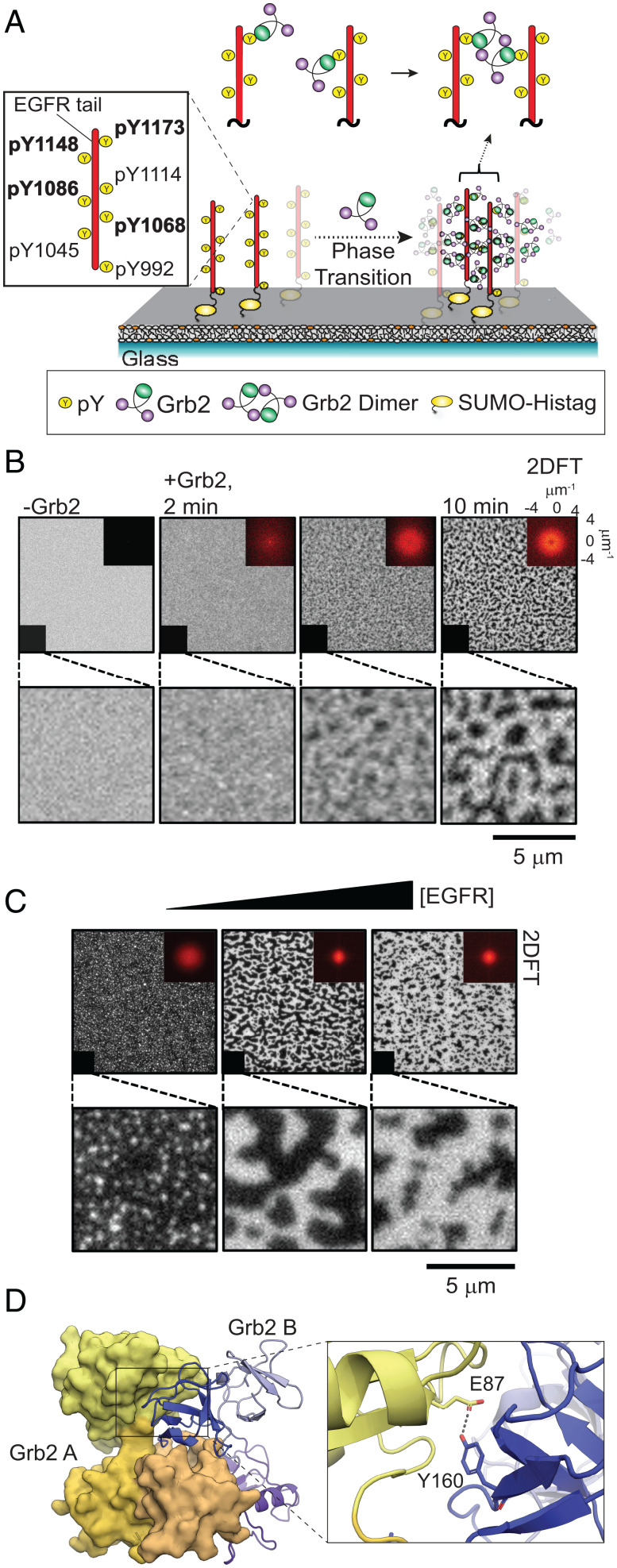
EGFR^TAIL^ phase transition induced by the dimerization of Grb2. (*A*) The schematic of the reconstitution experiment. The phosphorylated EGFR^TAIL^ is tethered on the supported membrane. EGFR^TAIL^ has several tyrosine sites that can be phosphorylated, including Tyr-1068, Tyr-1086, Tyr-1148, and Tyr-1173, related to Grb2 recruitment. Grb2 is injected into the chamber and recruited by EGFR^TAIL^. Grb2 is shown to dimerize on the supported membrane. The dimerization of Grb2 drives the assembly of EGFR^TAIL^:Grb2 into phase transition. EGFR^TAIL^ is initially in the state where it is mobile on the supported bilayer (*Left*). EGFR^TAIL^ becomes part of the protein condensate after the phase transition is triggered (*Right*). (*B*) TIRF images of Alexa Fluor 488–labeled EGFR^TAIL^ on the supported bilayer, revealing a phase transition within 2 min after the addition of Grb2 to form a condensed phase of 950 molecules/μm^2^ density. The protein condensate of EGFR^TAIL^:Grb2 continued to mature over 10 min to achieve densities of ∼3,760 molecules/μm^2^. (*Bottom*) Magnified images of the lower-left corner in the images in the top row. (*Top*) The inserts in the upper right corners are the two-dimensional Fourier transform (2DFT) of the TIRF images, which confirm that the structure of the condensate is isotropic. (*C*) The macroscopic fractional area coverage of the EGFR^TAIL^:Grb2 condensate, as well as its morphological features, scale with the density of EGFR^TAIL^. Image data here, spanning from 50 to 3,000 molecules/μm^2^, illustrate EGFR^TAIL^:Grb2 condensate variation from small clusters (*Left*) to large interconnected domains (*Right*). (*D*) The crystal structure of Grb2 shows the asymmetric dimer (PDB:1GRI). The tyrosine 160 at the dimeric interface is illustrated in the magnified picture.

**Fig. 2.**
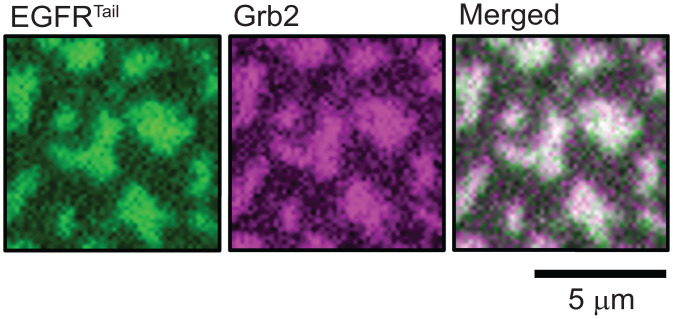
The colocalization between EGFR^TAIL^ and Grb2 after EGFR^TAIL^:Grb2 phase transition is achieved. Unlabeled Grb2 mixed with Alexa Fluor 647–labeled Grb2 (0.2 μM; labeling efficiency, 63%) was added to phosphorylated Alexa Fluor 488–labeled EGFR^TAIL^ to induce the phase transition of EGFR^TAIL^:Grb2. The TIRF images were taken 10 min after the addition of Grb2. The first image (green) is from EGFR^TAIL^. The second image (magenta) is from Grb2. The third image is the merged image from EGFR^TAIL^ and Grb2.

Upon addition of Grb2, the EGFR^TAIL^ underwent a robust condensation phase transition, as illustrated schematically in Fig. 1*A*. Images tracking the phase transition process (Fig. 1*B*) show an initially homogeneous distribution of the EGFR^TAIL^, which underwent a coarsening process after injection of Grb2 (6 µM) to form a dense phase (bright in these images) interspersed with a sparse phase containing a very low density of monomeric EGFR^TAIL^. The domains exhibited undulating shapes and a sharp discontinuity in EGFR^TAIL^ density at the boundaries of the two phases. The Grb2-driven condensation of EGFR^TAIL^ occurred over a range of initial densities of the tails to form condensed phases of similar density but different overall area coverage. At lower EGFR^TAIL^ initial density, isolated circular EGFR^TAIL^:Grb2 condensates were observed, while fully percolating domain patterns were observed at higher densities (Fig. 1*C*). The presence of both Grb2 and EGFR^TAIL^ in the condensates was confirmed by separately labeling them with Alexa Fluor 488 (EGFR^TAIL^) and Alexa Fluor 647 (Grb2), respectively, and performing two-color imaging (Fig. 2).

**Fig. 3.**
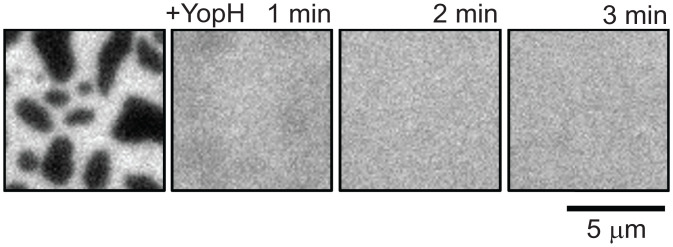
EGFR^TAIL^:Grb2 phase transition is reversed by phosphatase. (*Left*) TIRF image of EGFR^TAIL^ condensation taken at 15 min after the addition of Grb2 to phosphorylated EGFR^TAIL^ on the supported bilayer. (*Middle*) Image taken after the addition of 1 μM phosphatase, YopH. EGFR^TAIL^:Grb2 condensate is rapidly dissolved in 1 min. (*Right*) Image taken at 3 min after the addition of YopH shows uniformly distributed EGFR^TAIL^ (*SI Appendix*, Fig. S1).

The EGFR^TAIL^:Grb2 condensation is reversible and can be dispersed by addition of phosphatase. Addition of the phosphatase YopH (1 μM YopH and 6 μM Grb2 in bulk solution) rapidly disrupts the EGFR^TAIL^:Grb2 condensate, returning the system to a homogeneous and mobile distribution of EGFR^TAIL^ within 1 min (see Fig. 3). This confirms that even in the condensed state, sufficient dynamic unbinding and rebinding between Grb2 and the phosphorylated tyrosine sites on EGFR enables ready access by phosphatases [similar dynamic behavior has been observed in the LAT protein condensates (5)].

**Fig. 4.**
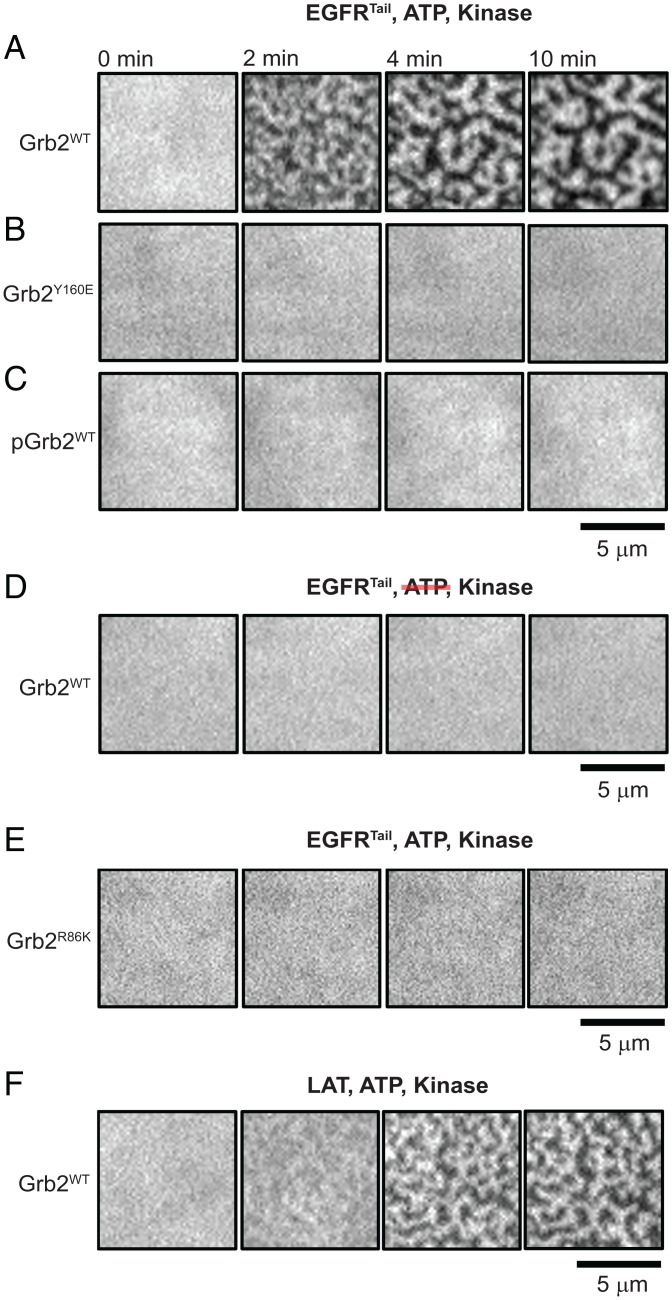
Grb2 dimerization as the key driving force to induce phase transition. TIRF images of EGFR showing the reconstitutions of (*A*) wild-type (WT) Grb2 and EGFR^TAIL^. Phase transition is observed at 3 min. (*B*) Grb2^Y160E^ and EGFR^TAIL^. EGFR^TAIL^ remains uniformly distributed. Phase transition is not detected. (*C*) pGrb2 and EGFR^TAIL^. Phase transition is not detected. (*D*) WT Grb2 and EGFR^TAIL^ without ATP. Phase transition is not detected. (*E*) Grb2^R86K^, SH2-impaired Grb2 mutant, and EGFR^TAIL^. No phase transition is observed. (*F*) WT Grb2 and LAT. Phase transition of LAT:Grb2 is observed in 6 min. (*A*–*F*) Hck was the kinase used to phosphorylate EGFR^TAIL^.

Formation of the EGFR^TAIL^:Grb2 protein condensates requires a multivalent crosslinking interaction. In previous studies of the LAT:Grb2:SOS protein condensate, SOS was presumed to be the primary crosslinking element. In the experiments described here, with no SOS present, we hypothesized that crosslinking must be occurring through Grb2 directly. The crystal structure of Grb2 reveals a dimeric assembly (PDB 1GRI) and Grb2–Grb2 interaction has been reported to play a functional role in FGFR signaling (35). In the case of FGFR signaling, phosphorylation at Y160 on Grb2 breaks the Grb2–Grb2 interaction and enables downstream signaling. The Grb2 Y160 residue is located in the Grb2 dimer interface and a Y160E Grb2 mutant (Grb2^Y160E^) has been reported to block dimerization (36). To test if a Grb2 dimer interaction is responsible for the EGFR^TAIL^:Grb2 condensation, we characterized the condensation with the Grb2^Y160E^ as well as phosphorylated wild-type Grb2 (pGrb2). Experimentally, pGrb2 was phosphorylated by Hck, and further analysis by mass spectrometry confirmed phosphorylation at Y160 (*SI Appendix*, Table S1). In both cases, EGFR^TAIL^:Grb2 condensation was fully disrupted (Fig. 4 *A*–*C* and *SI Appendix*, Fig. S2). A Grb2 dimer interface serves as the crosslinking element in the EGFR^TAIL^:Grb2 condensate and this interaction can be negatively regulated by phosphorylation of Grb2 at Y160. Control experiments without ATP confirmed that the EGFR^TAIL^:Grb2 condensate is dependent on EGFR phosphorylation (Fig. 4*D*). Additionally, a Grb2^R86K^ mutant [which has a disrupted SH2 domain (46)] could not induce condensation (Fig. 4*E*), further affirming the essential role of Grb2–SH2 binding to phosphorylated tyrosine residues on EGFR^TAIL^. Finally, we confirmed the Grb2^Y160E^ mutant is recruited to phosphorylated EGFR^TAIL^, even though it is incapable of driving condensation, confirming Grb2^Y160E^ retains a functional SH2 domain (*SI Appendix*, Fig. S2).

The condensate formed by Grb2 and the EGFR^TAIL^ is analogous to that formed by Grb2 and LAT, and we speculated that the same Grb2-mediated effects are also present in LAT condensates. Experiments with LAT and Grb2 confirmed that a LAT:Grb2 condensate also forms without the addition of SOS or other crosslinking elements, in a manner very similar to what we have observed with the EGFR^TAIL^:Grb2 system. (Fig. 4*F*).

### EGFR Condensation Enhances Ras Activation by SOS.

**Fig. 5.**
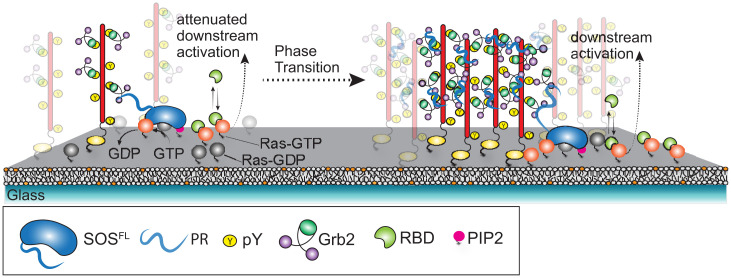
The schematic of downstream signaling modulated by SOS^PR^-titrated EGFR^TAIL^:Grb2 phase transition. SOS^PR^ was used as the strong crosslinker to induce the phase transition of the molecule assembly, EGFR^TAIL^:Grb2:SOS, at different levels. To initiate the downstream signaling, Grb2, SOS^PR^, GTP (1 mM), Alexa Fluor 555–labeled SOS^FL^, and Alexa Fluor 647–labeled RBD (50 nM) were added together to the bilayer. Once SOS^FL^ was activated, the activated SOS^FL^ would stay on the membrane and processively activate Ras. The activated Ras (Ras-GTP) is detected by RBD. The downstream signaling at the activation of Ras was read by the fluorescence intensity of RBD on the bilayer. Also see Ras activation in *Materials and Methods*.

To measure the effects of EGFR condensation on downstream signaling, we reconstituted the signaling pathway from EGFR to Ras. This reconstitution is depicted schematically in Fig. 5 and includes the EGFR^TAIL^, Grb2, phosphatidylinositol-4,5 bisphosphate (PIP_2_) lipid (which participates in SOS autoinhibition release), full-length SOS (SOS^FL^), Ras, and a fluorescently labeled construct of the Ras binding domain of Raf RBD), which provides a final readout of Ras activation by its selective recruitment to RasGTP (10). Ras is anchored to the membrane via coupling the C181 residue in the Ras hypervariable region to maleimide lipids (PE MCC), following well-established methods (47–49). Prior to any experiments, membrane-associated Ras was loaded with guanosine diphosphate (GDP) to establish a starting condition of fully inactive Ras (*Materials and Methods*).

In these experiments, Grb2 and the SOS^FL^ protein were maintained at sufficiently low concentrations that their presence alone was insufficient to trigger a condensation phase transition. To establish facile experimental control of the phase transition, we titrated in the PR domain of SOS (SOS^PR^; residues 1051 to 1333). SOS^PR^ is a strong crosslinker of Grb2, which has been used to control the LAT:Grb2:SOS condensation (10), and behaves similarly with the EGFR^TAIL^. Unlike SOS^FL^, SOS^PR^ lacks the catalytic domains and thus cannot contribute to Ras activation. To initiate the Ras activation reaction, SOS^FL^ (Alexa Fluor 555 labeled), Grb2, and SOS^PR^ were added in various ratios together with guanosine triphosphate (GTP; 1 mM) and Alexa Fluor 647–labeled RBD (50 nM) to the prephosphorylated EGFR^TAIL^ and RasGDP on the supported membrane. Subsequently, Grb2 bound to the phosphotyrosine sites on the EGFR^TAIL^, recruiting both SOS^FL^ and SOS^PR^, and a lateral protein condensation phase transition proceeded to various degrees depending on the specific protein concentrations in each experiment. At the membrane, full-length SOS further engaged PIP_2_ through its pleckstrin homology domain and Ras (in the GDP or GTP state) through its allosteric binding site (50, 51), ultimately releasing autoinhibition and beginning to catalyze the nucleotide exchange reaction of RasGDP to RasGTP. RasGTP levels were read out in real time through the selective binding of Alexa Fluor 647–labeled RBD to RasGTP. We use a modified RBD construct that exhibits fast and reversible binding kinetics (10) and achieves ∼5-s time resolution for tracking RasGTP.

**Fig. 6.**
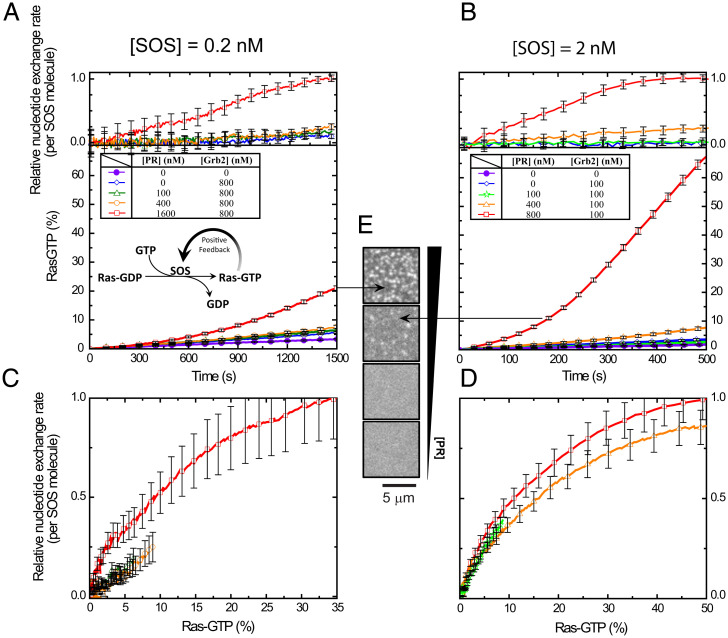
Ras activation modulated by EGFR^TAIL^:Grb2 phase transition. (*A*) The kinetic traces of Ras activation and the relative nucleotide exchange as the function of time at an SOS concentration [SOS] of 0.2 nM. (*B*) The kinetic traces of Ras activation and the relative nucleotide exchange as the function of time at [SOS] of 2 nM. (*C*) The plot of the relative nucleotide exchange rate against the activated Ras showing the positive feedback of SOS at [SOS] of 0.2 nM. (*D*) The plot of the relative nucleotide exchange rate against the activated Ras showing the positive feedback of SOS at [SOS] of 2 nM. [Grb2], concentration of Grb2; [PR], concentration of SOS^PR^.

Ras activation trajectories were tracked as a function of the EGFR^TAIL^ condensation state for two different, overall SOS^FL^ concentrations (0.2 and 2 nM). Data plotted in Fig. 6 *A* and *B* illustrate the overall Ras activation trajectories as well as the calibrated, per-molecule SOS activities for each condition. Representative images of the corresponding EGFR^TAIL^ condensation state are presented in Fig. 6*E*. For both SOS^FL^ concentrations, titrating in SOS^PR^ led to an abrupt enhancement in Ras activation after macroscopic condensation was achieved, as seen by the sharp divergence of the fastest trajectory (red) from trajectories for the other conditions in each case. This enhancement of Ras activation in EGFR^TAIL^ condensates was observed in the calibrated, per-molecule, SOS activities (Fig. 6 *A* and *B*, *Top*) as well as in overall reaction trajectories (*Bottom*). Thus, in addition to recruitment of more SOS to the condensates (e.g., through multivalent avidity effects), each SOS^FL^ molecule also activated more Ras over the same time compared with uncondensed state of the EGFR^TAIL^. Also prominent in the fastest overall reaction trajectories from each SOS^FL^ concentration was RasGTP-driven positive feedback, which is evidenced by the positive curvature in the trajectories (20, 41, 52). In these reactions, a constant catalytic rate from SOS^FL^ would have resulted in a Ras activation trajectory with strictly negative curvature as progressively less RasGDP would have been available to be activated. The use of SOS^PR^ to titrate the EGFR^TAIL^ condensation was for experimental convenience only. As demonstrated in *SI Appendix*, Fig. S3, the EGFR^TAIL^ condensation, controlled exclusively through Grb2, was sufficient to modulate Ras activation by SOS^FL^ in a similar manner as observed with SOS^PR^. We have also experimentally demonstrated that the small amounts of SOS^PR^ used here were insufficient to drive condensation if the Grb2^Y160E^ mutant was used—thus a Grb2-Grb2 interaction appears to be essential under these conditions (*SI Appendix*, Fig. S4).

Further insight into how the EGFR condensate may be facilitating SOS activation of Ras can be obtained by examining the reaction rate as a function of RasGTP density. Plots of the relative overall nucleotide exchange rate as a function of RasGTP density for each condition in Fig. 6 *A* and *B* are presented in Fig. 6 *C* and *D*. Strong rate enhancements with increasing RasGTP are evident in all cases, again revealing RasGTP-driven positive feedback. The EGFR condensate-driven enhancement in Ras activation, which is dominant in the time trajectories of these reactions, is much less obvious when rates are mapped to RasGTP levels. At the higher SOS^FL^ concentration (Fig. 6*D*), the EGFR condensate effect is essentially undetectable, with all trajectories nearly overlaying each other. At the lower SOS^FL^ concentration (Fig. 6*C*), a modest increase (roughly double) in reaction rate is evident for the condensed state (red) compared with the other conditions. In the related LAT condensate system, Ras activation by SOS has been shown to follow a first-passage time mechanism, in which the overall system kinetics are predominantly set by the time it takes to get the first few SOS molecules activated (53) (although effects of LAT condensation were not addressed in that study). Ras activation measurements presented here suggest EGFR condensation is impacting the initiation of Ras activation more so than it is modulating steady-state aspects of the reaction. More efficient activation of the first few SOS molecules allows the RasGTP-driven positive feedback to kick in earlier, leading to a substantial difference in overall Ras activation.

## Discussion

The protein condensate of the EGFR^TAIL^ described here exhibits striking similarities with the LAT protein condensate with respect to its phase transition properties as well as the way it impacts Ras activation. However, the context of the LAT protein condensation in response to TCR activation differs dramatically from EGFR signaling with respect to the copy numbers of molecules involved. T cells have near single-molecule antigen-sensing capabilities (54–57) and can activate with only few triggered TCRs. In contrast, EGFR stimulation often involves thousands or even millions of activated receptors per cell (58–60). Among molecules in the EGFR-MAPK pathway, SOS is expressed at very low levels and is likely a limiting component (60). Thus, unlike the LAT:Grb2:SOS condensate, an EGFR:Grb2:SOS condensate would seem implausible if SOS was a key crosslinking element that needed to be present in stoichiometric ratios with EGFR. Observations of a two-component EGFR:Grb2 condensate that we report here, with a Grb2-Grb2 interaction serving as the primary crosslinker, relieve SOS of its requisite crosslinking role. We are then free to envision physiological EGFR condensates, primarily scaffolded by Grb2–Grb2 interactions, which contain substantially substochiometric copy numbers of SOS relative to the numbers of EGFR molecules. EGFR condensation, nonetheless, exhibits significant control over the ability of SOS to activate Ras.

More broadly, the EGFR condensate establishes a layer of the EGFR signal transduction mechanism, proximal to ligand-mediated EGFR activation itself, that offers regulatory control over the efficiency of downstream signal propagation. EGFR condensation may be influenced by other physical and chemical parameters, such as local mobility of EGFR, membrane topographical features, or other molecules participating in the condensation (61–63). Additionally, the fact that Grb2-Grb2 binding is disrupted by Y160 phosphorylation reveals a potential active regulatory mechanism by which EGFR condensation could be curtailed. Since Grb2 in condensates is in constant exchange with cytosolic Grb2 (5), such a mechanism would require a cell-wide shift in Grb2 phosphorylation levels; it remains an open question if such regulation is utilized under physiological conditions. An intriguing side effect of Grb2 phosphorylation as a potential negative regulator is that tyrosine kinase inhibitors, such as those widely used as cancer therapeutics in EGFR-driven cancers (64), could also inhibit this negative regulatory mechanism—conceivably interfering with their desired effects.

## Materials and Methods

### Chemicals.

DOPC and 1,2-dioleoyl-sn-glycero-3-[(*N*-(5-amino-1-carboxypentyl)iminodiacetic acid)succinyl] (Ni^2+^-NTA-DOGS; nickel salt) were purchased from Avanti Polar Lipids. Alexa Fluor 488, Alexa Fluor 555, and Alexa Fluor 647 maleimide dyes were purchased from Thermo Fisher. Bovine serum albumin (BSA), (±)-6-hydroxy-2,5,7,8-tetramethylchromane-2-carboxylic acid, catalase, 2-mercaptoethanol (BME), NiCl_2_, H_2_SO_4_, and ATP were purchased from Sigma-Aldrich. Glucose oxidase was purchased from Serva. Tris(2-carboxyethyl)phosphine (TCEP), glucose and H_2_O_2_ were purchased from Thermo Fisher Scientific. MgCl_2_ was purchased from EMD Chemicals. Tris-buffered saline (TBS) was purchased from Corning.

### Protein Purification.

#### EGFR^TAIL^.

The C-terminal region of EGFR^TAIL^ (residues 1015 to 1210, where the signal peptide of EGFR is included in numbering) fused to a N-terminal His6 and a SUMO tag with a tobacco etch virus (TEV) cleaving site was cloned into pProEX-HTb. The plasmid was transformed into BL21(DE3) *Escherichia coli*. The transformed cells were grown at 37 °C in 1 L of Terrific Broth medium until an optical density at 600 nm (OD_600_) reached 0.7. The culture was induced with 1 mM isopropyl β-d-1-thiogalactopyranoside (IPTG) and allowed to grow overnight at 18 °C. After overnight expression, the culture was spun down for 15 min at 4,000 rpm and resuspended in 15 to 30 mL of Ni-NTA buffer (500 mM NaCl, 20 mM Tris⋅HCl at pH 8.5, 20 mM imidazole). The EGFR^TAIL^-expressing cells were lysed by sonication. The insoluble fraction of the lysate was separated by ultracentrifugation. The supernatant was applied to a HisTrap FF column (GE Healthcare). The EGFR^TAIL^ was eluted by eluting buffer (500 mM NaCl, 20 mM Tris⋅HCl at pH 8.5, 500 mM imidazole). The elution fractions were concentrated by an Amicon Ultra Centrifugal Filter Unit (30 kDa molecular weight cutoff [MWCO]; Millipore). The concentrated Grb2 was loaded onto an S75 30/300 column (GE Healthcare) equilibrated in Buffer A (150 mM NaCl, 20 mM Tris⋅HCl at pH 8.0, 1 mM TCEP). Finally, purified EGFR^TAIL^ was aliquoted and stored at −80 °C after flash freezing for further labeling reactions.

#### Grb2.

pET-28 plasmid containing full-length Grb2 fused to N-terminal His6 was transformed into BL21(DE3) *E. coli*. The transformed cells were grown at 37 °C in 1L Terrific Broth medium until OD_600_ reached 0.7. The culture was induced with 1 mM IPTG and allowed to grow overnight at 18 °C. After overnight expression, the culture was spun down for 15 min at 4,000 rpm and resuspended in 15 to 30 mL of Ni-NTA buffer (500 mM NaCl, 20 mM Tris⋅HCl at pH 8.5, 20 mM imidazole, 5% glycerol). The Grb2-expressing cells were lysed by sonication with the addition of 1 mM phenylmethylsulfonyl fluoride and 0.1 mM BME. The insoluble fraction of the lysate was spun down at 16,500 rpm for 45 min at 4 °C. The supernatant was applied to a HisTrap FF column (GE Healthcare). Grb2 was eluted by eluting buffer (500 mM NaCl, 20 mM Tris⋅HCl at pH 8.5, 500 mM imidazole, 5% glycerol). The elution fraction was loaded onto a desalting column equilibrated in Buffer A (150 mM NaCl, 20 mM Tris⋅HCl at pH 8.0, 1 mM TCEP, 5% glycerol). The fraction of the Grb2 peak was collected. Grb2 was incubated with TEV protease overnight to cleave the His tag. The cleaved Grb2 was reapplied to a HisTrap column. The flow-through was collected and concentrated by an Amicon Ultra Centrifugal Filter Unit (10 kDa MWCO). The concentrated Grb2 was loaded onto an S75 30/300 column (GE Healthcare) equilibrated in Buffer A. The fractions of Grb2 monomer were collected and concentrated. Grb2 was further equilibrated at 37 °C for 10 min to reestablish the equilibrium between the monomer and dimer. Finally, Grb2 was aliquoted and stored at −80 °C after flash freezing.

#### Ras.

H-Ras S118C containing residue 1 to 181 fused to N-terminal His_6_ was purified. The N-terminal His6 tag was removed by TEV protease. The procedures were described in the previous work (65).

#### SOS^FL^.

Full-length SOS protein was prepared via a split-intein approach. The N- and the C-terminal fragments of human SOS1 were expressed in BL21 (DE3) bacteria and purified separately. The two fragments with high purity were ligated by the intein reaction to generate SOS^FL^. The details of the design for the intein reaction, expression, and purification are described in ref. 10.

#### SOS^PR^.

pETM vector containing the open reading frame (ORF) His6-MBP-(Asn)10-TEV-SOS1 (residue 1051 to 1333, human) PR domain was transformed into BL21 (DE3) bacteria. The expression and purification are reported in ref. 5.

#### RBD-K65E.

pETM11 vector containing the ORF his6-GST-PreScission-SNAPtag-Raf1 RBD (residue 56 to 131, K65E) derived from the Raf-1 human gene was transformed into BL21 (DE3) bacteria. RBD was expressed and purified as described previously (10).

### Protein Labeling.

Proteins were first diluted to 100 µM. Alexa Fluor 488, 555, or 647 maleimide dye was dissolved in anhydrous dimethyl sulfoxide to make a 10 to 20 mM working stock. The dye was added to the protein within three-fold molar excess over protein concentration to avoid overlabeling. The reaction mixture was incubated at room temperature for 1 h. To quench the labeling reaction, dithiothreitol was added to the reaction mixture at the final concentration of 10 mM. Amicon Ultra Centrifugal Filter Unit was used to wash the free dye away by adding more buffer while concentrating the protein. The concentrated protein was loaded to an analytical-grade size exclusion column (S75; GE Healthcare) for further purification. The fractions of the purified protein were collected and concentrated. Finally, the labeled protein was aliquoted and stored at −80 °C after flash freezing.

### Supported Lipid Bilayers.

Supported lipid bilayers (SLBs) were formed by rupturing small unilamellar vesicles (SUVs). SUVs were made by mixing DOPC, Ni^2+^-NTA-DOGS, PE MCC, and PIP_2_ lipids (92:4:2:2 by molar percent) in chloroform. Chloroform of solution was evaporated using a rotary evaporator for 10 min at 40 °C to obtain the dried lipid films, which were further dried by purging N_2_ for 10 min. The vesicle solution was formed from the hydration of the dried lipids by H_2_O with the concentration of 1 mg/mL, using vortexing. SUVs were made by sonicating the vesicle solution for 90 s in an ice-water bath. SLBs were prepared on the glass substrate cleaned by piranha etch (glass coverslips, bottom thickness 170 µm ± 5 µm; Ibidi) combined with the flow chamber (sticky-Slide VI 0.4; Ibidi). The SUVs were mixed with 10 mM TBS buffer at pH 7.4 (1:1 ratio by volume) and injected into the chamber for 30 min of incubation to make SLBs. The incubation of 1 mg/mL BSA in TBS buffer for 10 min was used to block the defects in SLBs. H-Ras was anchored on PE MCC lipids through the maleimide chemistry by incubating 0.5 mg/mL H-Ras with SLBs for 3 h. The free PE MCC lipids were quenched by 5 mM BME. EGFR^TAIL^ and Hck were anchored on SLBs via his-tag–Ni^2+^NTA chemistry by incubating EGFR^TAIL^ and Hck at 150 and 10 nM for 30 min. During this step, 1 mM GDP was included to ensure that Ras was loaded with GDP. To phosphorylate the tyrosine sites of EGFR^TAIL^, 1 mM ATP and 5 mM MgCl_2_ in TBS were added to SLBs and incubated for 10 min. The mobility of the membrane-bound proteins was examined by fluorescent recovery after photobleaching. The densities of the membrane proteins were determined by the averaged intensities of the epifluorescence images via the calibration curve between the intensity of the image and the density of the protein measured by the FCS, described previously (47).

### TIRF Microscopy.

An Eclipse Ti inverted microscope (Nikon) with a TIRF system and iXon electron-multiplying charged-coupled device camera (Andor Technology) was used for imaging. TIRF microscopy was performed with a Nikon 100 × 1.49 numerical aperture oil-immersion TIRF objective, a TIRF illuminator, a Perfect Focus system, a motorized stage, and a U-N4S four-laser unit as a laser source (Nikon). The laser unit has the solid-state lasers for the 488 nm, 561 nm, and 640 nm channels and was controlled using a built-in acousto-optic tunable filter. The laser powers of 488 nm laser, 561 nm laser, and 640 nm laser were set to 5.2, 6.9, and 7.8 mW respectively measured with the field aperture fully opened. The 405/488/561/638 nm Quad TIRF filter set (Chroma Technology Corp.), along with supplementary emission filters of 525/50 m, 600/50 m, 700/75 m for 488 nm, 561 nm, 640 nm channels, respectively, were used. Images are acquired using the Nikon NIS-Elements software with the exposure time of 50 ms.

### Ras Activation and Imaging Analysis.

SOS^PR^ was used as the strong crosslinker to induce the phase transition of the molecular assembly, EGFR^TAIL^:Grb2:SOS, at different levels while the concentrations of Grb2 were kept constant, avoiding the bias in signal outputs due to the competition for Grb2. SOS^PR^ without the catalytic domain for the nucleotide exchange of Ras would not contribute to the Ras activation done by SOS^FL^. To initiate the downstream signaling, Grb2, SOS^PR^, GTP (1 mM), Alexa Fluor 555–labeled SOS^FL^, and Alexa Fluor 647–labeled RBD (50 nM) were added together to the bilayer. While the phase transition of EGFR^TAIL^ was occurring, SOS was recruited by Grb2. Once SOS^FL^ was activated, the activated SOS^FL^ would stay on the membrane and start to processively activate Ras. The activated Ras (Ras-GTP) on the membrane was further detected by RBD in the bulk solution via the interaction between Raf and activated Ras. The downstream signaling at the activation of Ras was read by the fluorescence intensity of RBD on the bilayer, using TIRF images. At the end of each Ras activation trace, 20 nM of the catalytic domain of SOS^FL^ was used to fully activate Ras on SLBs. The averaged intensity of the RBD TIRF image corresponding to total Ras molecules on SLBs was used as the normalization factor to calibrate different Ras densities over different traces. SOS^FL^ on the membrane was tracked at the same time as the fluorescence intensity of RBD on the bilayer was being monitored. The Ras activation by SOS^FL^ was allowed to interact directly with the phase transition of EGFR^TAIL^. The concentrations of Grb2 (800 nM and 100 nM) were chosen to modulate the recruitment of SOS^FL^ by Grb2, making the time scale of Ras activation comparable within 30 min between the two data sets of low and high concentrations of SOS.

Single-molecule images of SOS^FL^ were tracked by an ImageJ plugin, TrackMate (66), to obtain the number of activated SOS^FL^ molecules on the membrane. SOS^FL^ molecules were localized with the detector of difference of Gaussian. The initial diameter was set to 6 pixels. Typically, 50 to 3,000 molecules were detected per image. In this study, the Ras activation modulated by EGFR^TAIL^ phase transition at both low and high concentrations of SOS (0.2 and 2 nM) were interrogated separately. Dividing the instantaneous increase of activated Ras by the number of SOS molecules on the bilayer gave the relative nucleotide exchange rate of SOS as a function of time. To study the positive feedback of SOS^FL^, the relative nucleotide exchange rates of SOS^FL^ were plotted against activated Ras (percentage of Ras-GTP).

## Supplementary Material

Supplementary File

## Data Availability

All study data are included in the article and/or *SI Appendix*.
