# Supplementary material for: A two-component protein condensate of the EGFR cytoplasmic tail and Grb2 regulates Ras activation by SOS at the membrane
Source: Proc Natl Acad Sci U S A. 2022 May 4;119(19):e2122531119. doi: 10.1073/pnas.2122531119 (PMC9181613; doi:10.1073/pnas.2122531119)
Supplement: Supplementary File [file pnas.2122531119.sapp.pdf]

PNAS
www.pnas.org

Supplementary Information for
A two-component protein condensate of the EGFR cytoplasmic tail and Grb2 regulates Ras activation by SOS at the membrane.

Chun-Wei Lin[1,2,3], Laura M. Nocka[1,2,3], Brittany L. Stinger[1], Joseph B. DeGrandchamp[1], L.J. Nugent Lew[1], Steven Alvarez[1], Henry T. Phan[1], Yasushi Kondo[1,2,3], John Kuriyan[1,2,3,4,*], Jay T. Groves[1,5,6,*]

[1]Department of Chemistry, University of California, Berkeley, CA 94720, USA
[2]Department of Molecular and Cell Biology, University of California, Berkeley, CA 94720, USA
[3]California Institute for Quantitative Biosciences, University of California, Berkeley, CA 94720, USA
[4]The Howard Hughes Medical Institute
[5]Division of Molecular Biophysics and Integrated Bioimaging, Lawrence Berkeley National Laboratory, Berkeley, CA 94720
[6]Institute for Digital Molecular Analytics and Science, Nanyang Technological University, Singapore

*Corresponding Author: John Kuriyan, Jay T. Groves
Email: kuriyan@berkeley.edu (J.K.), jtgroves@lbl.gov (J.T.G.)

This PDF file includes:

Figures S1 to S4
Tables S1

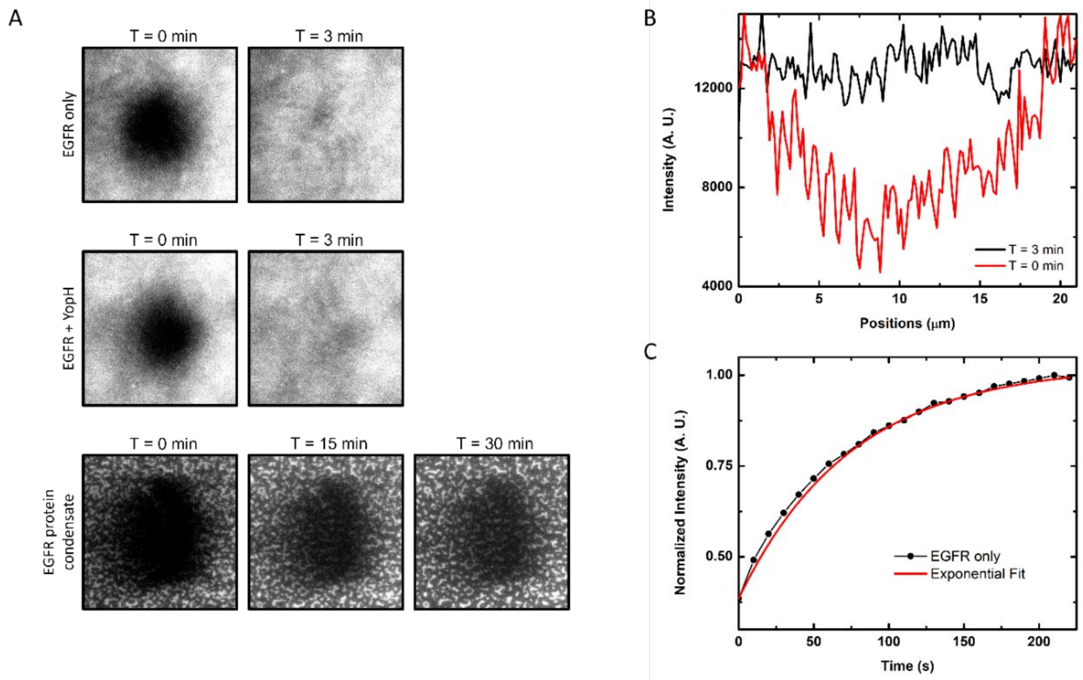

Fig. S1. FRAP experiments of EGFR. (A) FRAP images. Top: $EGFR^{TAIL}$ on the bilayer, middle: $EGFR^{TAIL}$ after YopH treatment, bottom: $EGFR^{TAIL}$:Grb2 protein condensate. (B) Intensity profile of FRAP image from $EGFR^{TAIL}$ only at 0 and 3 min. (C) The recovery trace of $EGFR^{TAIL}$ only. The red solid line is the exponential fit where τ_D = 73 s.

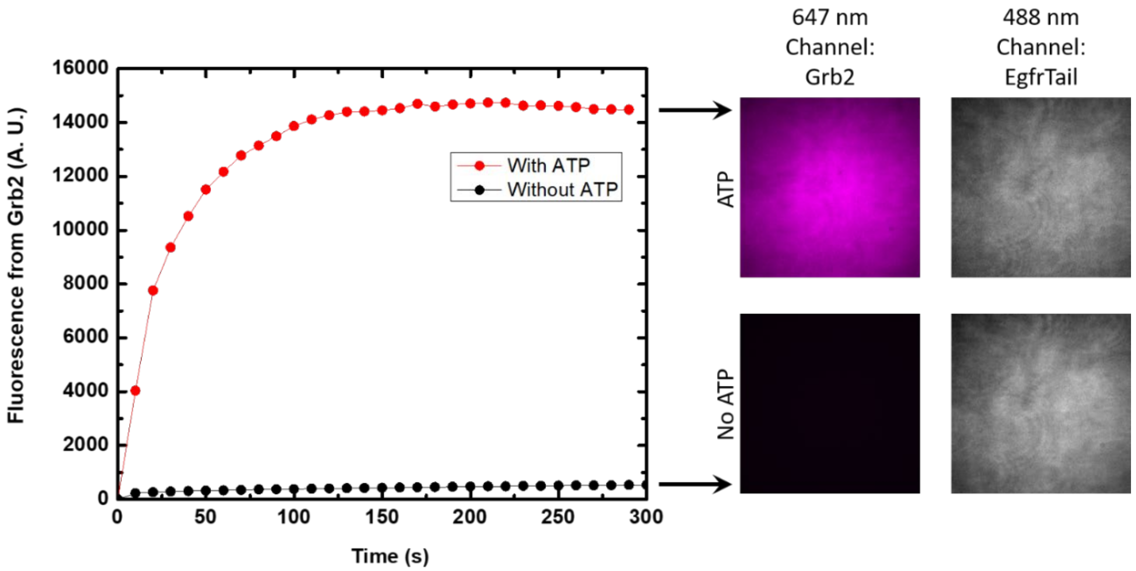

Fig. S2. Grb2^{Y160E} responses to the phosphorylation of EGFR^{TAIL}. Alexa Fluor 647 labeled Grb2^{Y160E} (6 μM, labeling efficiency of 48%) is added to phosphorylated EGFR^{TAIL} on the bilayer. The averaged fluorescence intensity from the TIRF images of Grb2^{Y160E} on the supported bilayer increases rapidly corresponding to the recruitment by EGFR^{TAIL} (the red curve in the left graph). The control experiment is shown by the black curve where EGFR^{TAIL} is not phosphorylated. The TIRF images from Grb2^{Y160E} (in magenta) and EGFR^{TAIL} (in gray) taken 10 minutes after the addition are shown at the right side of the figure. No phase transition is observed using Grb2^{Y160E}.

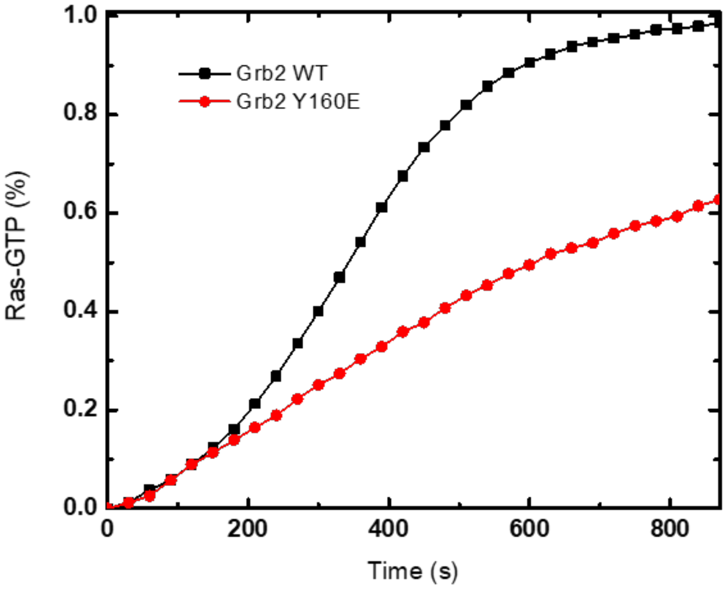

Fig. S3. Ras activation between wildtype Grb2 and Grb2^{Y160E}. In this Ras activation assay, the crosslinker, SOS^{PR} is not used. To initiate the downstream signaling, Grb2 (200 nM), GTP (1 mM), Alexa Fluor 555 labeled SOS^{FL} (4 nM) and Alexa Fluor 647 labeled RBD (50 nM) are added together to phosphorylated EGFR^{TAIL} on the supported bilayer. The activated Ras (Ras-GTP) is detected by RBD. Also see Ras activation in Materials and Methods.

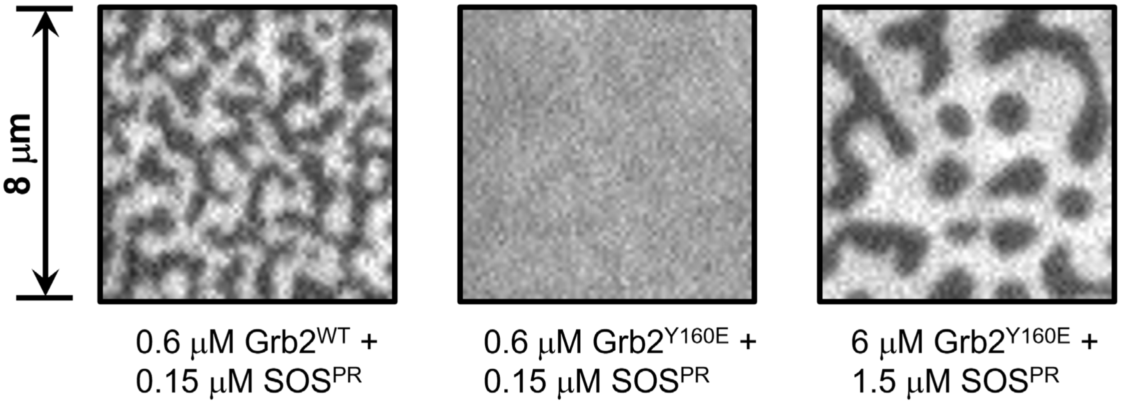

Fig. S4. TIRF images of Alexa Fluor 488 labeled EGFR^{TAIL} on the supported bilayer 10 minutes after the addition of Grb2 and SOS^{PR} showing small amounts of SOS^{PR}, such as were used in our detailed studies of the effect of EGFR condensation on SOS activity, are insufficient to drive condensation if the Grb2^{Y160E} mutant is used—thus a Grb2:Grb2 interaction appears to be essential, at least under those conditions. At high enough SOS^{PR} levels, it is possible to overcome the need for the Grb2:Grb2 interaction in reconstituted experiments.

Sequence	Modifications	Charge	MH+ [Da]	m/z [Da]	Note
VLNEEcDQNWYK	C6(Carbamidomethyl)	2	1597.69658	799.35193	Tyr 37
VLNEEcDQNWyKAELNGK	C6(Carbamidomethyl); Y11(Phospho)	2	2289.98516	1145.49622	Tyr 37
VLNEEcDQNWYKAELNGK	C6(Carbamidomethyl)	3	2210.01670	737.34375	Tyr 37
NyIEMKPHPWFFGK	Y2(Phospho)	3	1873.84989	625.28815	Tyr 52
NYIEMKPHPWFFGK		2	1793.88445	897.44586	Tyr 52
DIEQVPQQPTyVQALFDFDPQEDGELGFR	Y11(Phospho)	3	3461.55979	1154.52478	Tyr 160
DIEQVPQQPTYVQALFDFDPQEDGELGFRR		3	3537.69577	1179.90344	Tyr 160
DIEQVPQQPTYVQALFDFDPQEDGELGFR		3	3381.59543	1127.87000	Tyr 160

Table S1. Three tyrosines of Grb2 show the phosphorylation by Hck. Grb2 has seven tyrosines. The peptide analysis of mass spectrum covers 85% of Grb2 sequence which includes six tyrosines in Grb2. The phosphorylated Grb2 sample was digested by trypsin overnight before the analysis by the mass spectrometer.
